# Paediatric versus adult trauma car accidents mortality in a northern Italy trauma system

**DOI:** 10.1186/1757-7241-21-S1-S2

**Published:** 2013-05-28

**Authors:** A Giugni, L Giuntoli, F Del Corso, F Mengoli, A Volpi, E Bigi, M Menarini, A Maioli, M Grazia, C Simonetti, I Turriziani, A Chieregato, G Gordini

**Affiliations:** 1 Maggiore Hospital Emergency Department and Trauma Care, Bologna, Italy; 2 Maggiore Hospital Anesthesia and intensive care unit, Bologna, Italy; 3 Maggiore Hospital Intensive Care Unit, Parma, Italy; 4 Bufalini Hospital Neuro-Intensive Care Unit, Cesena, Italy

## Background

Trauma accounts for a large proportion of childhood mortality. Few data exist about injury patterns within paediatric trauma in Italy. Recognizing high-risk patterns may help to improve care and outcome[1].

## Methods

Data from the 315 (<17 years old) major trauma cases collected in the Emilia-Romagna trauma registry (RRTG) since 2006 to 2010 were analyzed. The inclusion criteria are ISS>15 or Intensive Care Unit (ICU) admission, which is decided by clinical judgment without standard criteria. In Emilia Romagna there are no Paediatric Trauma Centers. The main outcome measure[2] was mortality at discharge from the ICU. Patients dead on arrival or early in the Emergency Room were not considered. Injury severity was coded according to the '98 AIS version.

## Results

There were 315 (90 cases/y) paediatric cases; mean age was 11.79±5.34 (median 14, IQR 95% 8) years. In the same period 3575 patients older than 16 years were included; mean age was 49.86±21.01 (median 47, IQR 95% 37) years. The overall mortality was 7.1% for children, and 12.9% for adults; the difference was significant (p=0.0005). The commonest mechanism of injury (MOI) was road traffic accident (RTA). Car crashes (CC) accounted for 30.2% of RTAs for patients under 17, and for 39.3% for those older than 16. Mortality for CC was 11.6% among children, and 9.0% among adults, with no difference (p=0.3913).

The mortality for CC is shown in Table 1, stratified by ISS and age.

**Table 1:** 

	ISS 1-15			ISS 16-24			ISS 25-40			ISS 41-75		
	deaths	total	mortality	deaths	total	mortality	deaths	total	mortality	deaths	total	mortality

**≥ 17 years**	8	165	4.8	22	549	4.0	41	480	8.5	51	200	25.5

**< 17 years**	0	18	0	0	28	0	4	34	11.8	7	14	50

A difference was observed for ISS 41-75 class; children mortality resulted significantly higher than the one of adults. (p=0.0373).

## Conclusion

Many severe RTAs injuries are caused by CC, both in children and in adults. The overall mortality is significantly higher in adults, while CC mortality is similar in the two groups, except for the most severely injured patients, where it is worse in the younger.

Some questions remain open, thus needing further investigations:

-are paediatric safety systems widely and properly used by adults when transporting children in their cars?;

-are children more exposed to lethal lesions often travelling as passengers, and being unaware of the imminent crash, consequently unprepared to counteract deceleration?

-is the ISS reliable for paediatric patients (who have a higher incidence of head injuries)?[3] This score assigns the same weight to all body regions, disregarding the importance of head injuries in mortality;

-are adult trauma centers prepared to deal with severely injured children, given their rare occurrence? Should caregivers usually working on adults receive an additional specific paediatric training?

Future analyses of RRTG may help to evaluate both outcomes and management of high- risk children, and to improve the quality of care.
